# Supplementary material for: Structures of the holo CRISPR RNA-guided transposon integration complex
Source: Nature. 2022 Nov 28;613(7945):775–82. doi: 10.1038/s41586-022-05573-5 (PMC9876797; doi:10.1038/s41586-022-05573-5)
Supplement: Supplementary file 1 — Supplementary Figs. 1–11; Table 1, which includes all the oligonucleotides used in this study; and references. [file 41586_2022_5573_MOESM1_ESM.pdf]

nature portfolio

https://doi.org/10.1038/s41586-022-05573-5

Supplementary information

Structures of the holo CRISPR RNA-guided transposon integration complex

In the format provided by the authors and unedited

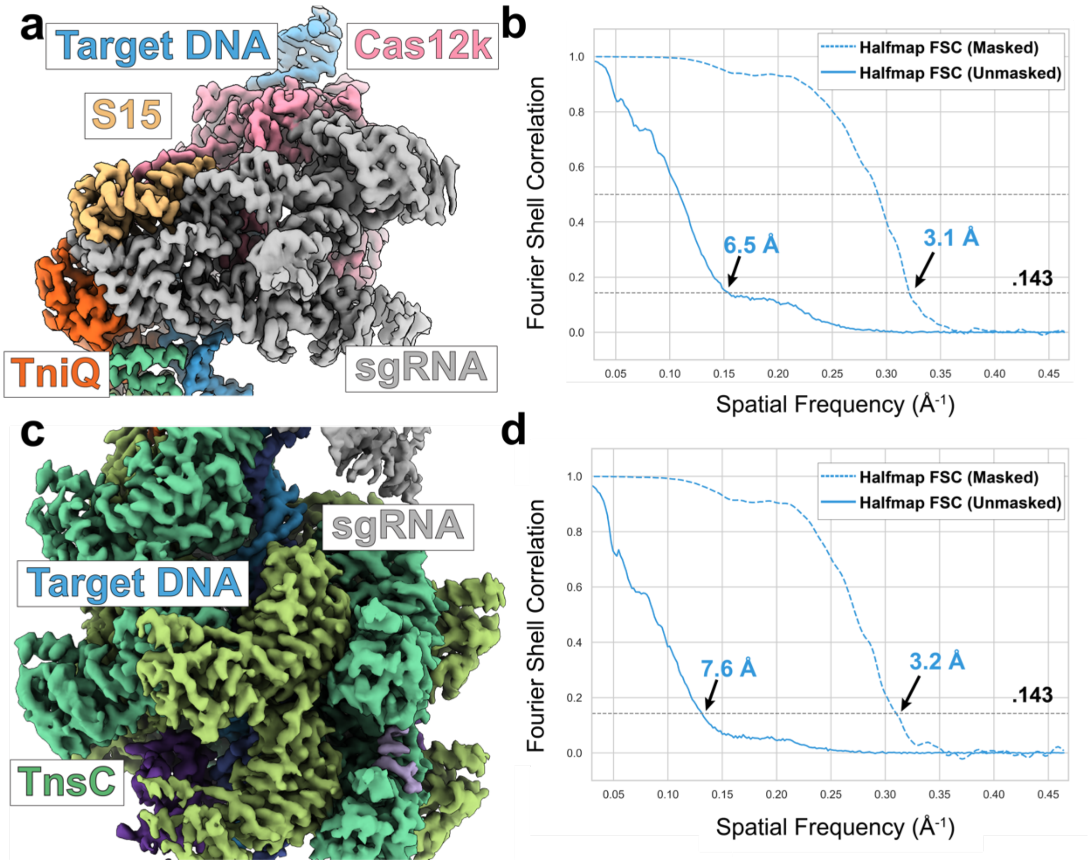

Supplementary Figure 1 | High-resolution cryo-EM reconstruction of local refinements. a. The cryo-EM reconstruction from the local refinement focused on the Cas12k region. colored according to previously defined colors (see Main Text, Fig. 1). **b.** Fourier shell correlation (FSC) curve from the local refinement of Cas12k region. Masked (dashed) or unmasked (solid) gold standard half-map FSC (blue) are shown for the refined reconstruction. Gold-standard FSC cutoff (0.143) is indicated with a dashed line and an estimated resolution based on this cutoff is indicated. **c.** Cryo-EM map from the 3D refinement focused on the TnsC region of the ShCAST transpososome. The sections of the transpososome that are not included in the refinement mask, including sgRNA (grey), are by comparison less well resolved. **d.** FSC curve of the local refinement cryo-EM reconstruction of the TnsC region of the major configuration.

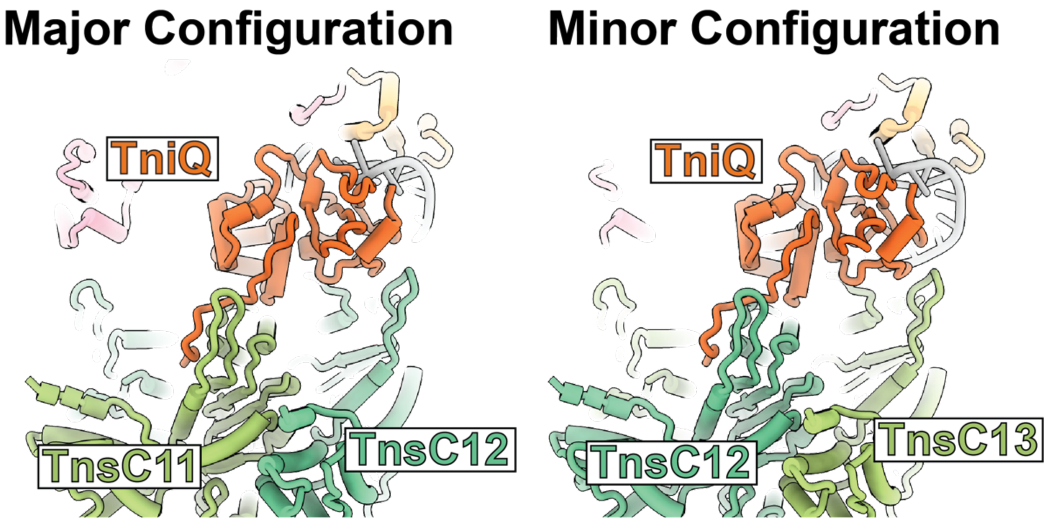

Supplementary Figure 2 | TniQ interacts with two TnsC protomers proximal to Cas12k in both major and minor configurations. In both major (left) and minor (right) configurations, TniQ bridges the two TnsC protomers closest to Cas12k in an identical manner. Colors for the ShCAST components are the same as previously defined in Fig. 1.

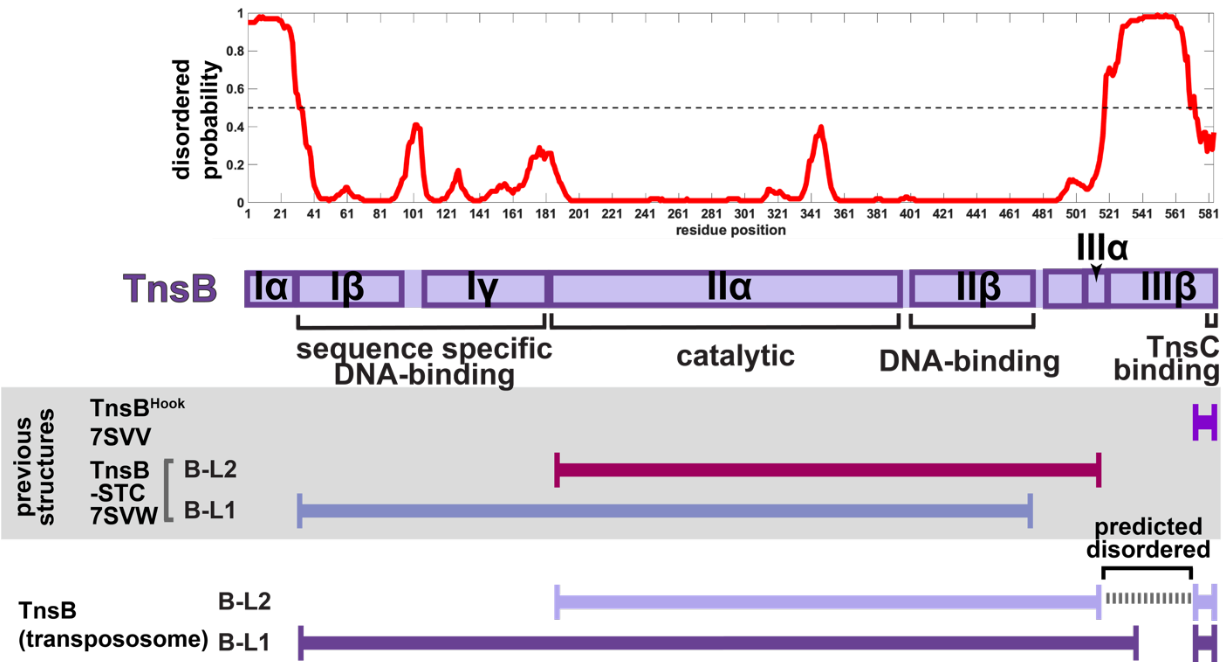

Supplementary Figure 3 | TnsB disordered prediction and modeled segments. Top panel: The probability of disorder at each residue position was predicted using DISOPRED3[27], and plotted. 1 indicates high probability of disorder, and 0 indicates low probability of disorder. Dashed line indicates 50% probability, the cutoff above which to consider a residue position to be predicted disordered. The purple diagram below the disordered prediction is the domain diagram of TnsB, along with the assigned functions for each domain. Domain names follow that of MuA. Gray box indicates the previous structures of TnsB resolved; The TnsB^{Hook} peptide corresponds to the last fifteen residues of TnsB, 570-584 (PDB: 7SVV). B-L1 and B-L2 are the two named conformations of TnsB resolved in the strand-transfer complex reported previously (PDB: 7SVW), also solved by cryo-EM. Bottom panel indicates the regions of B-L1 and B-L2 resolved within the transpososome. The stretch of residues corresponding to the flexible linker connecting the structured core of TnsB to the C-terminal TnsB^{Hook} is indicated in dashed gray lines.

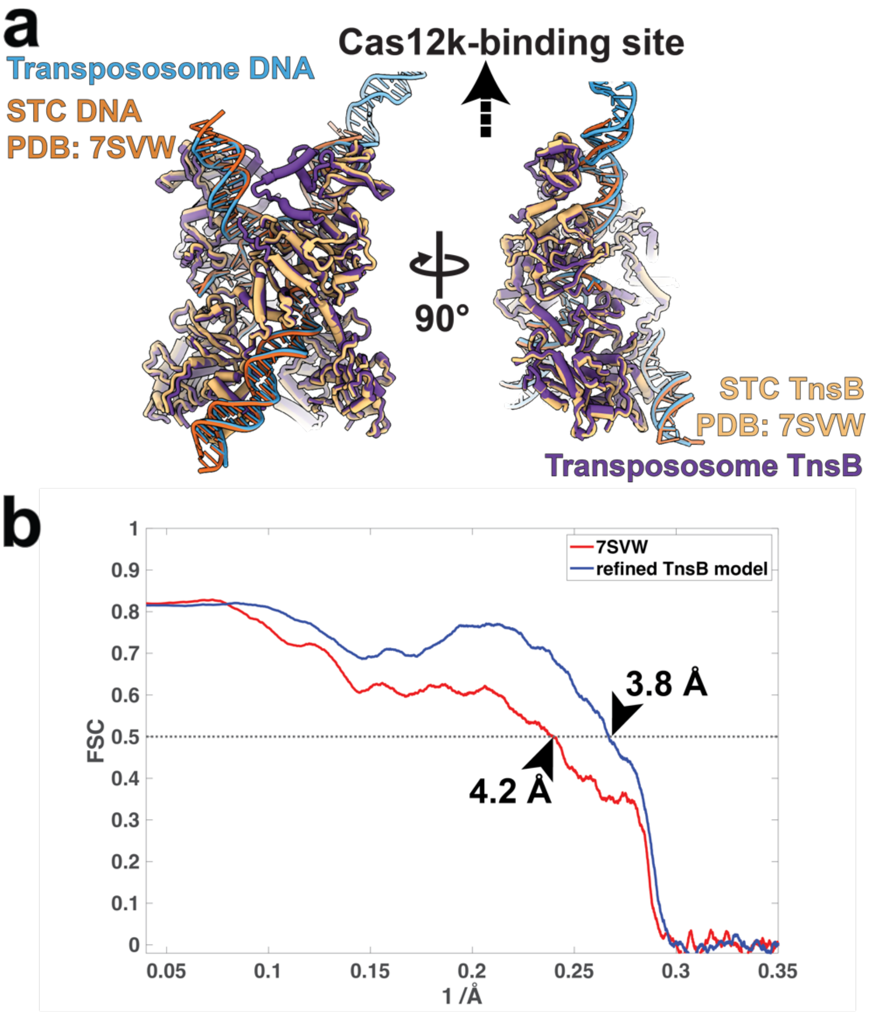

Supplementary Figure 4 | TnsB structure in the ShCAST transpososome is approximated well by the previously determined TnsB strand-transfer complex. a. The refined TnsB atomic model (this paper, purple) is superimposed onto the previous TnsB strand-transfer complex (STC) shown in tan (PDB: 7SVW). Overall architecture of the complexes is identical with the Ca r.m.s.d. of 1.54 Å. Target-DNA and transposon DNA (light blue) also appear structurally similar to the TnsB STC (orange). Two different orthogonal views are shown for comparison. The direction of the Cas12k-bound target-site is indicated with arrow. **b.** The masked model-map Fourier-shell correlation (FSC) curves are shown for the rigid-body docked TnsB STC (PDB: 7SVW, red curve) and the refined TnsB model (blue curve). A cutoff of 0.5 (dashed line) indicates the estimated resolution, or 'goodness of fit' of the atomic model against the map. The map used here is the locally refined TnsB cryo-EM reconstruction. Estimated resolution of each model is indicated with arrows. As expected, molecular refinement improved upon the starting model (PDB: 7SVW) from 4.2 Å to 3.8 Å, and roughly corresponds to the resolution of the locally refined cryo-EM map (3.2 Å).

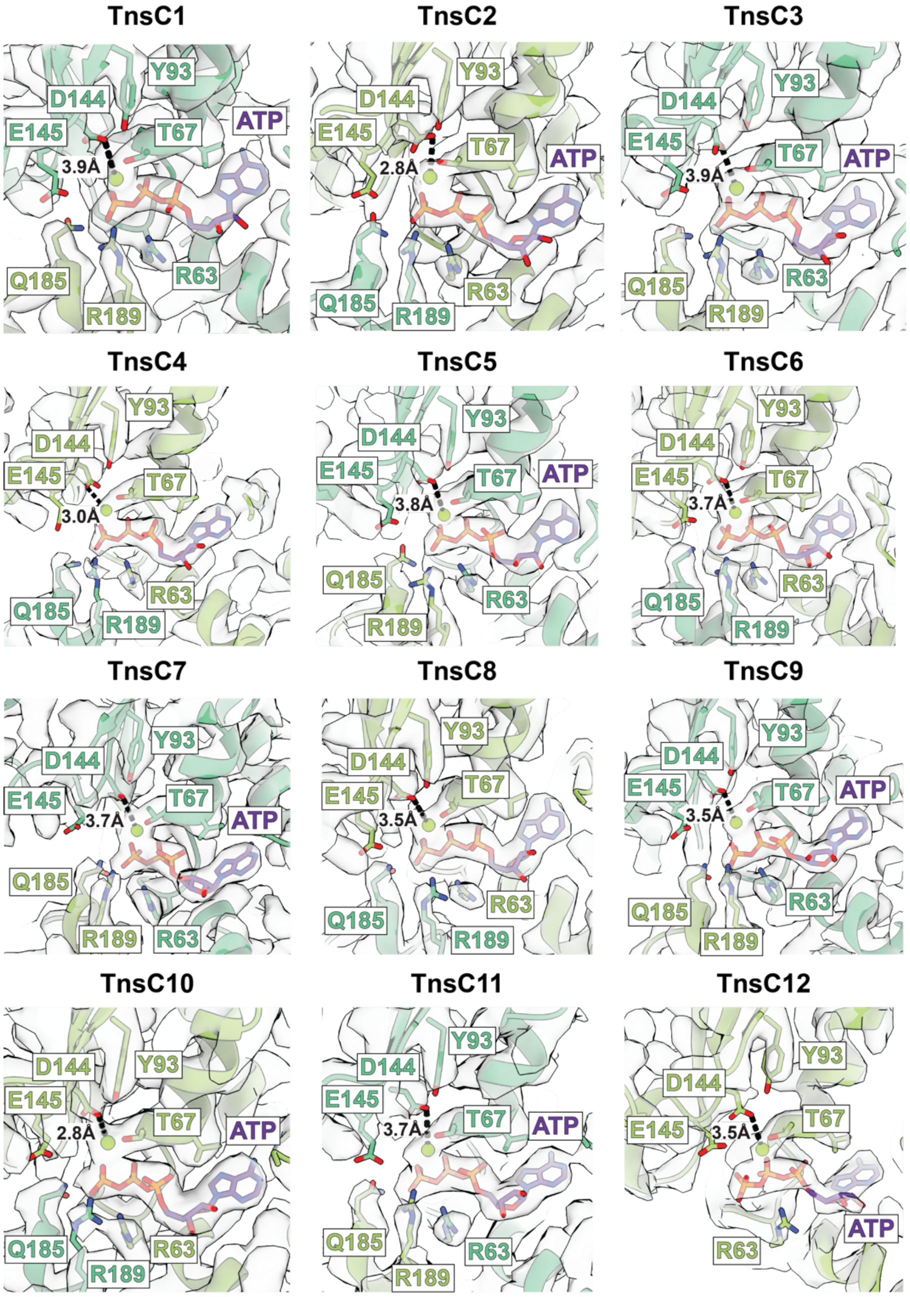

Supplementary Figure 5 | ATP is bound at all TnsC protomers in the ShCAST transpososome. ATP (purple) is bound at each of the TnsC protomers (numbered 1 through 12) in the ShCAST transpososome. Each of the ATP binding pockets is shown, docked into the cryo-EM density map (transparent gray surface). Magnesium is shown as a green sphere, and relevant sidechains are shown in stick. Because the ATP binding pocket sits between two TnsC protomers, either light green or dark green is used to shade the particular sidechains displayed, depending on which protomer (and therefore which ATP-binding pocket) is being shown. E145 and D144 are the catalytic residues, and T67 coordinates ATP-binding. Q185 and R189 are the sensor residues from the neighboring TnsC protomer that complete the ATP binding pocket. Distance from D144 to Magnesium is labeled in Å, and the interaction is shown with dashed lines.

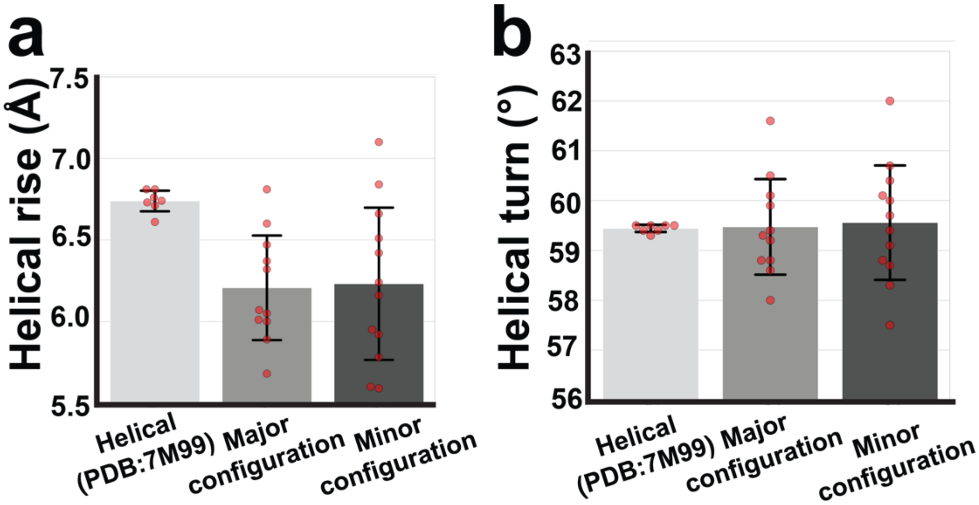

Supplementary Figure 6 | Measured helical parameters of the major and minor configurations of transpososomes are more variable compared to the helical TnsC structure. Average helical rise (**a.**) and helical turn (**b.**) estimated between TnsC protomers within ATPγS-bound helical TnsC (PDB: 7M99), TnsC from major configuration and minor configuration of transpososome. Helical parameters were estimated using Rosetta suite (see Methods for detail), and averaged to plot a bar-graph. Error bar indicates standard deviations from the estimated values. Both configurations of the transpososome have lower values of helical rise, and comparable values of helical turn. Large standard deviations of both helical rise and turn suggest that TnsC in context of the transpososome does not follow defined helical symmetry.

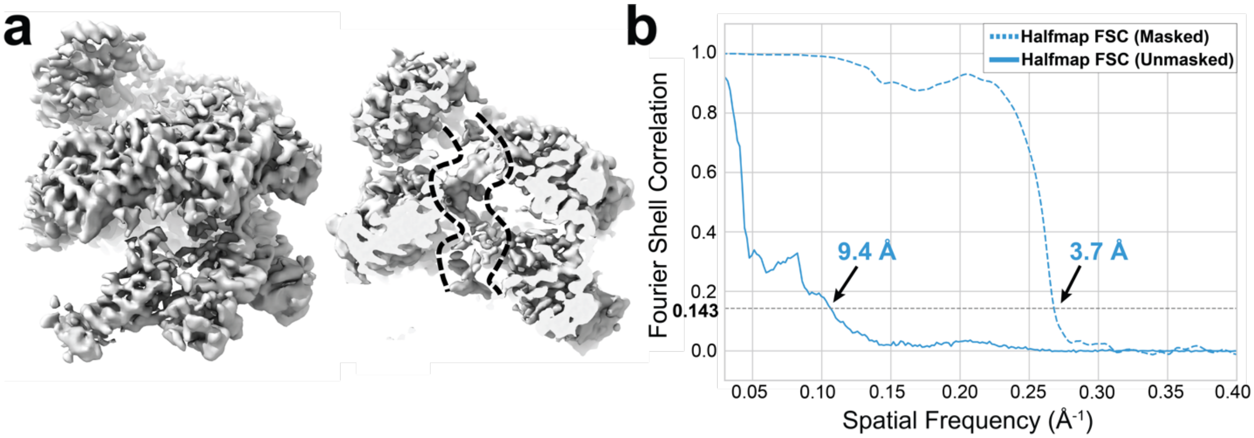

Supplementary Figure 7 | Reconstruction of the two turns of TnsC in transpososome using helical reconstruction approaches results in a lower-resolution map. a. Helical Cryo-EM reconstruction of TnsC in the major configuration. Helical parameters of the ATPγS-bound TnsC filaments (rise = 6.82 Å and twist = 60º) were used to impose helical symmetry. Local search of the helical parameters was allowed (see Methods for detail). The reconstruction (left) yields a lower resolution and a map with aberrant features, where the density of DNA in the map (right, indicated by dashed lines) is barely visible, compared to the result of local refinement (3.2 Å. Fig. S12). These results indicate that the TnsC protomers in transpososome does not follow the helical symmetry of TnsC filaments. **b.** Fourier shell correlation (FSC) curve of the helical refinement cryo-EM reconstruction of the TnsC region of the major configuration. Masked (dashed) or unmasked (solid) gold standard half-map FSC (blue) are shown for the refined reconstruction. Gold-standard FSC cutoff (0.143) is indicated with dashed line and estimated resolution based on this cutoff is indicated.

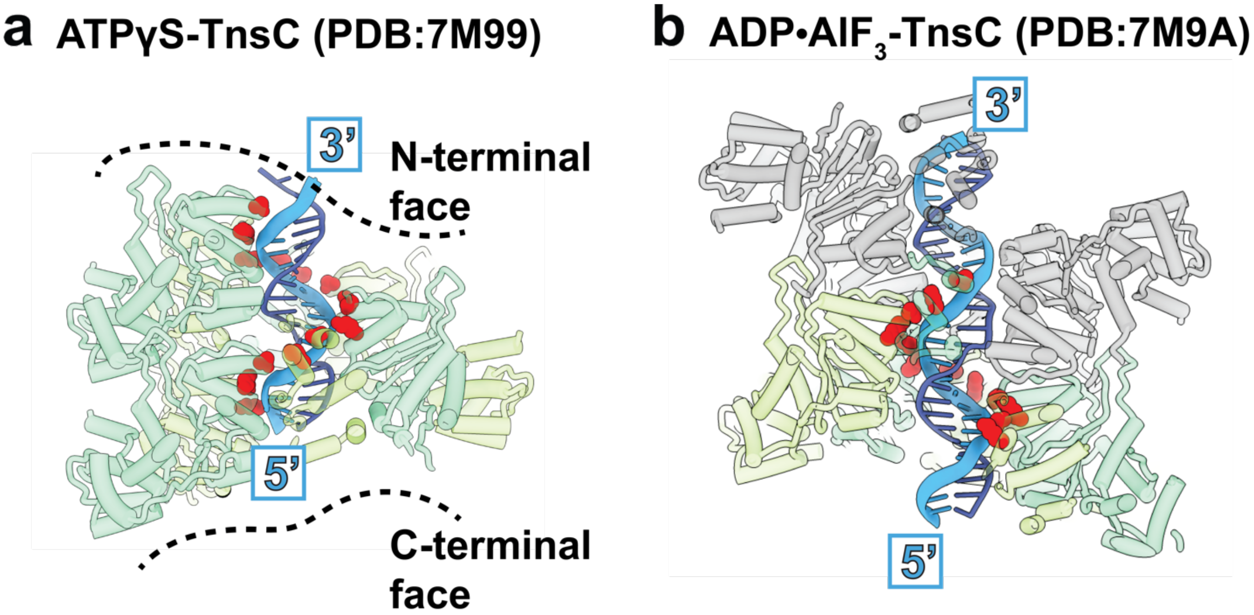

Supplementary Figure 8 | Previous TnsC helical filament structures selectively interact with one strand of DNA. a. The previously determined structure of ATPγS bound-TnsC (PDB: 7M99) is shown. TnsC (green) is bound to the DNA duplex (blue). Importantly, TnsC residues K103 and T121 (red spheres) track one strand of DNA (light blue, shown in thicker width) in the 5’ to 3’ direction going in the direction of TnsC’s C-terminal to N-terminal face (dashed black lines). The 5’ and 3’ end of the DNA strand interacting with TnsC are labeled. **b.** ADP·AlF_3 bound-TnsC (PDB: 7M9A) shows two hexamers, oriented in opposite directions. For clarity, the hexamer shown in the same orientation as in panel A is shown in green, and the other hexamer is shown in gray. DNA strand interacting with the green TnsC protomers is represented in light blue with thicker width.

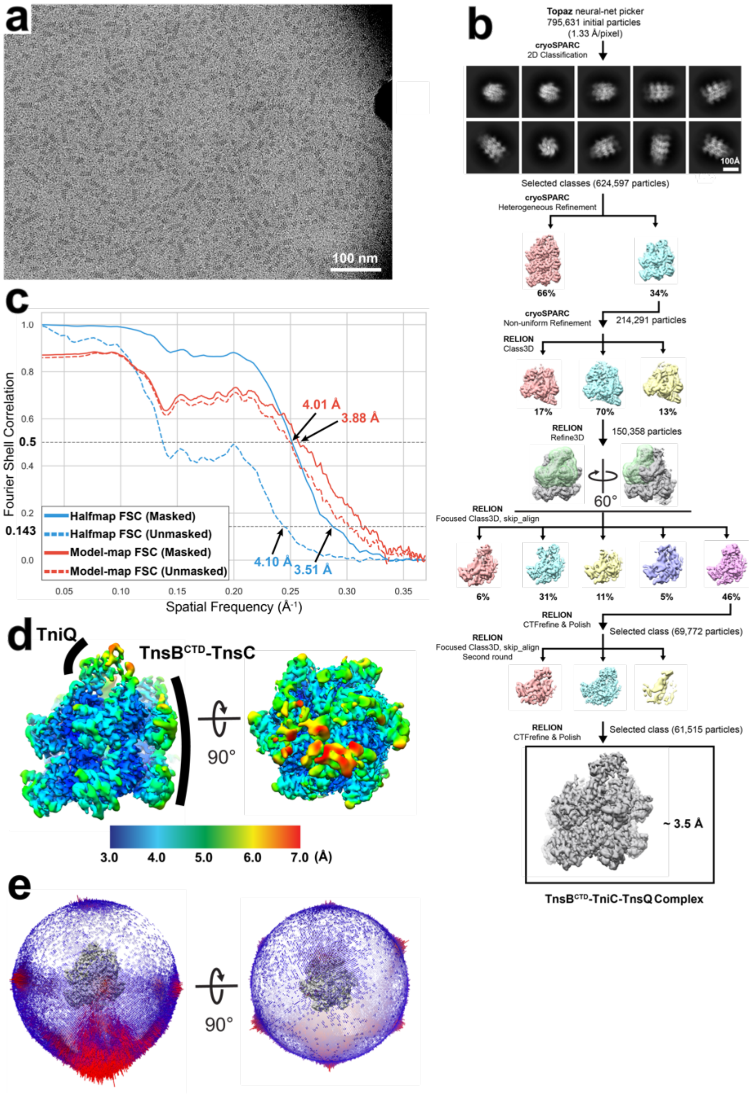

Supplementary Figure 9 | Image processing pipeline for TnsB^{CTD}-TnsC-TniQ structure. a. Representative cryo-EM micrograph of the TnsB^{CTD}-TnsC-TniQ sample. TnsB^{CTD} corresponds to TnsB residues from 476 to 584. White scale bar represents 100 nm. The micrograph shown is an example image from a dataset containing 1,271 micrographs. **b.** Image processing workflow of TnsB^{CTD}-TnsC-TniQ dataset. From initial 2D classification, classes with high-resolution 2D averages were selected for heterogeneous refinement in cryoSPARC[35]. Class with two turns of TnsC (cyan, 214,291 particles) was subjected to RELION[36,37] 3D classification, which resulted in a class with better cryo-EM density of TniQ (cyan, 150,358 particles). This particle stack was further subjected to two subsequent rounds of focused classification without alignment using the mask (green, overlaid on the grey input reconstruction) that includes one TniQ and two TnsC protomers. This resulted in the final particle stack of 61,515 particles. Final 3D refinement in RELION converged to a final ~ 3.5 Å reconstruction of the TnsB^{CTD}-TnsC-TniQ complex. **c.** Fourier-shell correlation (FSC) curve from gold-standard FSC (blue), and model-map FSC (red). Masked and unmasked FSC were represented in solid and dashed line respectively. The reported resolution was estimated based on the appropriate cutoff (0.143 for half-maps, and 0.5 for model-map FSC), and indicated on the plot. **d.** Local resolution maps from the final reconstruction of TnsB^{CTD}-TnsC-TniQ complex. Legend indicates corresponding resolutions of the surface color. TniQ region and TnsB^{CTD}-TnsC region of the reconstruction are indicated. **e.** Euler angle distribution of the final reconstruction. Red bars indicate the population of particles in a specific orientation.

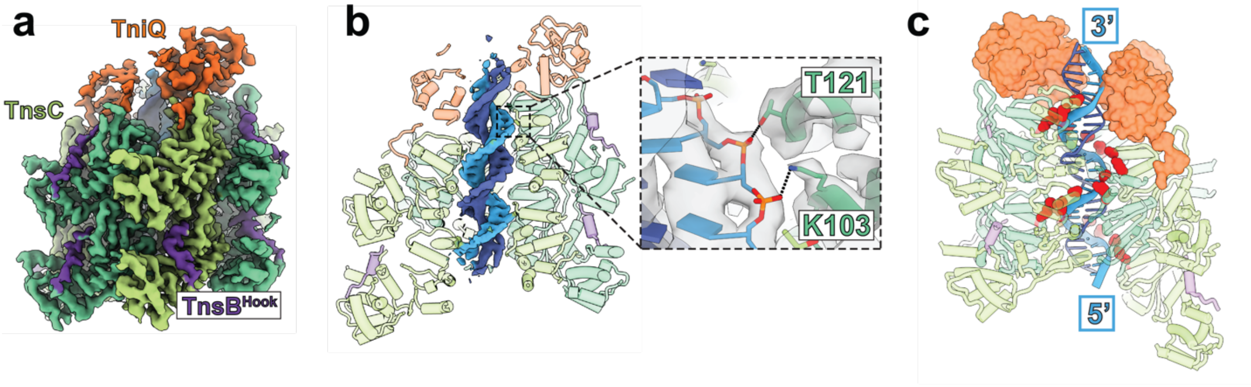

Supplementary Figure 10 | High-resolution cryo-EM reconstruction of TnsB^{CTD}-TnsC-TniQ shows TnsC interacts with DNA in the same fashion as previous TnsC structures. a. High-resolution cryo-EM structure of TnsB^{CTD}-TnsC-TniQ complex. TnsB^{Hook}, TnsC, and TniQ are represented in purple, green, and orange, respectively. **b.** Cryo-EM structure of the TnsB^{CTD}-TnsC-TniQ complex has high enough resolution to reveal atomic interactions between TnsC (green) and DNA (blue). DNA is shown as segmented density from the full map while TnsB^{Hook}, TnsC and TniQ are shown in ribbons. Close-up view on the right shows residues K103 and T121 from TnsC are close enough to form interactions (shown with dashed black lines) with the DNA sugar-phosphate backbone (blue, sticks). Both DNA and the residues fit well into the cryo-EM density (gray, transparent). **c.** TniQ (orange surface) subunits are bound to the N-terminal face of TnsC (green ribbon). Residues K103 and T121 from TnsC interact with one strand of DNA (light blue, shown in thicker width), consistent with previous TnsC structures. Residues K103 and T121 are shown in red spheres. The 5’ and 3’ ends of the DNA strand interacting with TnsC are labeled.

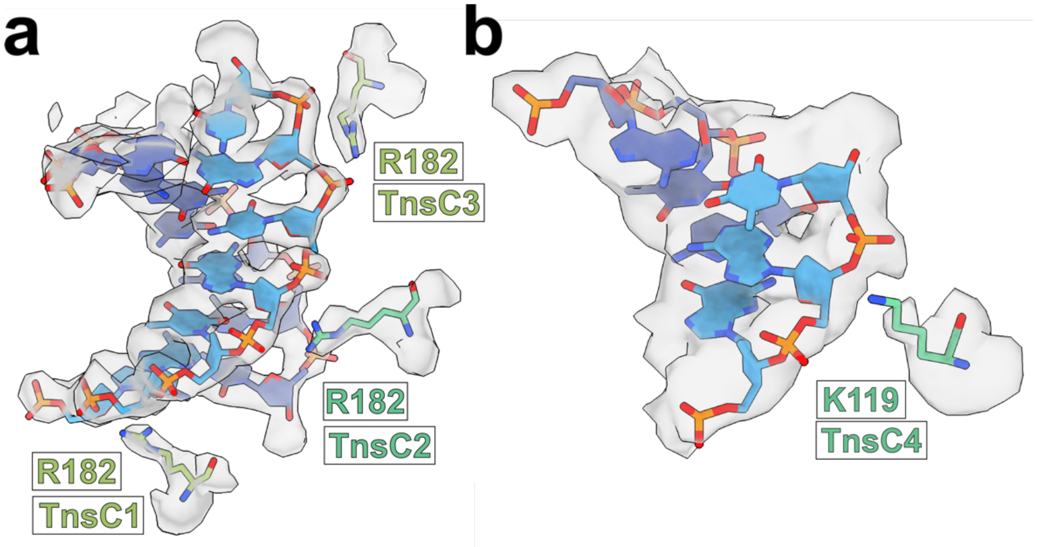

Supplementary Figure 11 | Cryo-EM density supports the newly identified interactions between TnsC and DNA in transpososome. a. Residue R182 in TnsC1-TnsC3 is positioned toward the sugar-phosphate backbone of DNA and is supported by cryo-EM density. Zoned map of the TnsC local refinement map is transparent. The map is zoned around the region containing both DNA strands and R182, shown in filled and stick representation, respectively. **b.** Residue K119 in TnsC4 is positioned toward the sugar-phosphate backbone of DNA and is supported by cryo-EM density. Zoned map of the TnsC local refinement map is transparent. The map is zoned around the region containing both DNA strands and K119, shown in filled and stick representation, respectively.

Oligonucleotides

Name	Description	Sequence	Used Figure(s)
RPS15_Ext_Hifi_F	Amplify S15 from genome, forward strand	GAACAGATTGGTGGATCCTCTCTAAGTACTGAAGCAACAGCTAA	1
RPS15_Ext_Hifi_R	Amplify S15 from genome, reverse strand	CTCGAGTGCGGCCGCTTAGCGACGCAGACCCAG	1
sgRNA_For	Cloning DNA template for sgRNA-PSP1 forward primer	GAATTAATACGACTCACTATAGGGATATTAATAGCGCCGCAAT	1
sgRNA_PSP1_Rev	Cloning DNA template for sgRNA-PSP1, reverse primer	CAGTGGCCTTATTAAATGACTTCTCCTTTCAACCCATTTAGGG	1
Target-LE_F_61bp	Transpososome assembly of 61bp spacing	GTCACAATGACATTAATCTGTCACCGACGACAGATAATTTGTCACTGTACAGTAGAATATAGATGCGCATCTATATAGATGCAAATTGAGTGGCCTTATTAAATGACTTCTCAACCAGTCAGCACGCCCAGACCAGGGCAC	1
Non-target_R_61bp	Transpososome assembly of 61bp spacing	GCGTGCTGACTGGTTCTCTTCAGTATTAATAAGGCCACTCAATTTGCATCTATATAGATGCGCATCTATAT	1
LE_R	Transpososome assembly	TGTACAGTGACAAATTATCTGTCGTCGGTGACAGATTAATGTCATTGTGAC	1
RE_F	Transpososome assembly	TTACTGATGACAATAATTTGTCACAACGACATATAATTAGTCACTGTACATCTACGATACGTAGCGGCCGACGCG	1

RE_R1	Transposome assembly	TGTACAGTGACTAATTATATGTCGTTGTGACAAATTATTGTCATCAGTAA	1
RE_R2	Transposome assembly	CGCGTCGGCCGCTACGTATC	1
LUEGO	Transposome assembly	GTGCCCTGGTCTGG	1
TniQ_Ndel_For	Cloning TniQ Δ1-10, forward primer	CTGTTCCTGATCAAGCCCTACGAG	2D
Ndel_primer_Rev	Cloning N-terminal deletions of both TniQ and TnsB, reverse primer	GGATCCACCAATCTGTTCTCTGTG	2D, 3E
TniQ_W10A_F12A_For	Cloning TniQ W10A+F12A, forward primer	GGCGCTGATCAAGCCCTACGAGGG	2D
TniQ_W10A_F12A_Rev	Cloning TniQ W10A+F12A, reverse primer	AGGGCAGGCTTCACGTCAGGGGC	2D
TniQ_H34A_For	Cloning TniQ H34A, forward primer	ACGGGCCAATGCGCTGTCTGCCTC	2D
TniQ_H34A_For	Cloning TniQ H34A, reverse primer	CTGAATCTGCCCAGGAAG	2D
BCQ_ top	$TnsB^{CTD}$-TnsC-TniQ complex assembly	GCTTGAAGCGGCGCACGAAAAACGC	SF9-10
BCQ_ bot	$TnsB^{CTD}$-TnsC-TniQ complex assembly	AACGCTTTCGCGTTTTTCGTGCGCCGCTTCA	SF9-10

TnsB_CTD_For	Cloninig TnsB CTD, forward primer	GGACTCGAAACAGAACAACTGGC	3E
TnsB_IIβ+CTD_For	Cloninig TnsB IIβ+CTD, forward primer	CTGATGAAGCAGAGCAGACGG	3E
TnsB_ΔCTD_For	Cloninig TnsB ΔCTD, forward primer	GGACTCGAAACAGAACAACTGGC	3E
TnsB_ΔCTD_Rev	Cloninig TnsB ΔCTD, reverse primer	CTGGGCGTGAGCCCTGGT	3E
TnsB_R432A_For	Cloning TnsB R432A, forward primer	CCTGATGTACGCAGGCGAGTACC	3E & 3F
TnsB_R432A_Rev	Cloning TnsB R432A, reverse primer	TTCTGGAACTGCAGACAG	3E & 3F
TnsB_Y439A_For	Cloning TnsB Y439A, forward primer	CCTGGCTGGAGCAGCCGGCGAGAC	3E & 3F
TnsB_Y439A_Rev	Cloning TnsB Y439A, reverse primer	TACTCGCCCCGGTACATC	3E & 3F
60bp_top	ATP-hydrolysis assay	CGACAGCTCCTCCATGAAAGCAGTGGCCTTATTAAATGACTTCTCAACCAGTCAGCACGC	3E
60bp_bot	ATP-hydrolysis assay	GCGTGCTGACTGGTTGAGAAGTCATTTAATAAGGCCACTGCTTTCATGGAGGAGCTGTCG	3E
TnsC_K103A_For	Cloning TnsC K103A, forward primer	AGGTCTCAGGGGCCACATTTTTGGT	4F

TnsC_K103A_Rev	Cloning TnsC K103A, reverse primer	AGGTCTCACCCCGCGGATTTGTTTAAAAAGATTACTGAGTACCTC	4F
TnsC_T121A_For	Cloning TnsC T121A, forward primer	AGGTCTCACCCTTTTGTTACCCGATACTTG	4F
TnsC_T121A_Rev	Cloning TnsC T121A, reverse primer	AGGTCTCAAGGGGCGGTATCTGATTTTCGAGATAGGACGA	4F
TnsC_R182A_For	Cloning TnsC R182A, forward primer	CGTGATCAAGGCAGACGAACAGGTG	4F
TnsC_R182A_Rev	Cloning TnsC R182A, reverse primer	GCATCCAGTCTATCGGTG	4F

Supplementary Table 1. Oligonucleotides used in this study

References

27 Jones, D. T. & Cozzetto, D. DISOPRED3: precise disordered region predictions with annotated protein-binding activity. *Bioinformatics* **31**, 857-863 (2015). https://doi.org:10.1093/bioinformatics/btu744

35 Punjani, A., Rubinstein, J. L., Fleet, D. J. & Brubaker, M. A. cryoSPARC: algorithms for rapid unsupervised cryo-EM structure determination. *Nat Methods* **14**, 290-296 (2017). https://doi.org:10.1038/nmeth.4169

36 Scheres, S. H. RELION: implementation of a Bayesian approach to cryo-EM structure determination. *J Struct Biol* **180**, 519-530 (2012). https://doi.org:10.1016/j.jsb.2012.09.006

37 Scheres, S. H. Processing of Structurally Heterogeneous Cryo-EM Data in RELION. *Methods Enzymol* **579**, 125-157 (2016). https://doi.org:10.1016/bs.mie.2016.04.012
